# Quality of life and mood assessment in conservatively treated cavernous malformation‐related epilepsy

**DOI:** 10.1002/brb3.2595

**Published:** 2022-04-25

**Authors:** Laurèl Rauschenbach, Pauline Bartsch, Alejandro N. Santos, Annika Lenkeit, Marvin Darkwah Oppong, Karsten H. Wrede, Ramazan Jabbarli, Witold X. Chmielewski, Börge Schmidt, Carlos M. Quesada, Michael Forsting, Ulrich Sure, Philipp Dammann

**Affiliations:** ^1^ Department of Neurosurgery and Spine Surgery University Hospital Essen Essen Germany; ^2^ Institute for Medical Informatics Biometry and Epidemiology University Hospital of Essen Essen Germany; ^3^ Department of Neurology, Division of Epilepsy University Hospital Essen Essen Germany; ^4^ Department of Diagnostic and Interventional Radiology and Neuroradiology University Hospital Essen Essen Germany

**Keywords:** anxiety, cerebral cavernous malformation, depression, epilepsy, quality of life

## Abstract

**Background:**

To estimate the quality of life, anxiety, depression, and illness perception in patients with medically treated cerebral cavernous malformation (CCM) and associated epilepsy.

**Methods:**

Nonsurgically treated patients with CCM‐related epilepsy (CRE) were included. Demographic, radiographic, and clinical features were assessed. All participants received established questionnaires (short‐form 36 health survey, SF‐36; hospital anxiety and depression score, HADS‐A/D; visual analogue scale score, VAS) assessing the functional and psychosocial burden of disease. To some extent, calculated values were compared with reference values from population‐based studies. Test results were related to seizure control.

**Results:**

A total of 37 patients were included. Mean age was 45.8 ± 14.4 years, and 54.1% were female. Diagnosis of CRE was significantly associated with attenuated quality of life and increased level of anxiety, affecting physical and psychosocial dimensions. The assessment of illness perception identified considerable burden. HADS was significantly associated with VAS and SF‐36 component scores. Efficacy of antiepileptic medication had no restoring impact on quality of life, anxiety, depression, or illness perception.

**Conclusions:**

CRE negatively influences quality of life and mood, independent of seizure control due to antiepileptic medication. Screening for functional and psychosocial deficits in clinical practice might be useful for assessing individual burden and allocating surgical or drug treatment.

## 1 INTRODUCTION

Cerebral cavernous malformations (CCMs) are the second most common type of neurovascular malformation in the central nervous system, representing between 10% and 15% of all vascular malformations (Washington et al., 2010). Although there is low blood pressure and slow blood flow within these lesions, CCMs carry considerable risk of intracerebral hemorrhage (Taslimi et al., 2016). Irrespective of bleeding occurrence, CCMs can implicate headaches, seizures, or focal neurological deficits (Horne et al., 2016). CCM‐related epilepsy (CRE) is a common phenomenon in CCM, with an incidence of ≈30% (Al‐Shahi Salman et al., 2012; Dammann, Wrede, et al., 2017; Horne et al., 2016). Even though the underlying mechanisms are poorly understood, few studies identified associations between CRE and CCM multiplicity, cortical involvement, or CCM‐related hemosiderin deposition (Dammann, Schaller, et al., 2017; He et al., 2017; Menzler et al., 2010; Moultrie et al., 2014). Notably, CCM treatment is still subject to debate. Evidence‐based treatment guidelines are scarce and decision‐making is often limited to clinical experience (Dammann et al., 2021; Rosenow et al., 2013). Although lesion removal in CRE patients seems justified in selected cases, brain surgery remains a delicate procedure, and noninvasive treatment approaches must be considered, particularly in patients who have minor or no functional impairments (Dammann, Wrede, et al., 2017).

Epileptic seizures can have a considerable impact on health‐related quality of life (HRQOL) (Mahrer‐Imhof et al., 2013), and treatment pathways should take into account not only neurological but also psychosocial burden. While the vast majority of CCM studies focus on different treatment strategies and risk factors leading to hemorrhage (Al‐Shahi Salman et al., 2016), literature reporting on HRQOL is rare (Bicalho et al., 2017; Rinkel et al., 2019). Recently published data highlight the impact of CCM disease on self‐reported quality of life but HRQOL and mood have never been studied exclusively in medically treated CRE patients (Herten et al., 2021).

In this study, conservatively treated patients were screened for disease‐related functional and psychosocial burden. Moreover, the results were compared to the average population and related to seizure control.

## 2 METHODS

### 2.1 Study design

A prospective cross‐sectional study was performed of all patients who were admitted to our specialized outpatient clinic for CCM disease between November 2017 and January 2020. During consultation, all patients were given general information about diagnosis, treatment strategies, and prognosis. Participants needed to fulfill the inclusion criteria mentioned below. Informed consent was obtained from all participants. The study was conducted in accordance with the principles expressed in the Declaration of Helsinki. A local ethics committee approved all procedures (Review Board Identification Number: 15‐6636‐BO). The trial was performed in accordance with Strengthening the Reporting of Observational Studies in Epidemiology protocol. Parts of the data were published previously in a study, investigating HRQOL in patients with untreated cavernous malformations of the central nervous system (Herten et al., 2021).

### 2.2 Inclusion and exclusion criteria

We included patients aged between 18 and 80 years and diagnosed with CRE according to the definition by Rosenow et al. (2013). CCM diagnosis was based on magnetic resonance imaging (MRI) including T1, T2, contrast‐enhanced, and susceptibility‐weighted or T2*‐weighted gradient echo imaging. Patients were treated conservatively. Individuals who received invasive treatment and were hospitalized within 3 months prior to study were excluded. Patients required sufficient knowledge of the German language to cope with the testing.

### 2.3 Data collection

Clinical data and imaging data were assessed prospectively and in accordance with CCM reporting standards (Al‐Shahi Salman et al., 2008). An experienced neuroradiologist evaluated imaging data. The following clinical and radiological features were assessed: age, sex, number of CCMs, lesion localization, occurrence of CCM‐related hemorrhage (Taslimi et al., 2016), physical or psychiatric comorbidities, epilepsy semiology, number of seizures, antiepileptic medication, time between first and last CCM‐related seizure, time between first CCM‐related seizure and neuropsychological testing, and time between last CCM‐related seizure and neuropsychological testing. The degree of disability was determined using the modified Rankin Scale (mRS). Outcome was considered favorable if the patient had a mRS less than or equal to 2 and unfavorable if the mRS was greater than 2. HRQOL was assessed in a standardized interview using the German version of the 36‐item short form health survey (SF‐36). Depression and anxiety were addressed using the hospital anxiety and depression rating scale (HADS‐A/D). Moreover, subjective perception of disease and burden of CRE were evaluated using a visual analog scale (VAS). Testing was conducted by one interviewer to avoid interobserver effects.

The SF‐36 questionnaire addresses two main domains comprised of 8 subdomains with 36 questions items: physical functioning (PF), social functioning (SF), role limitations due to physical problems (RP), role limitations due to emotional problems (RE, three items), mental health (MH, 5 items), vitality (VT, four items), bodily pain (BP, two items), and general health perceptions (GH, five items). Additionally, we determined two overall component scores: physical health component score (PCS) and mental health component score (MCS). For each subdomain, an overall score was assessed (0 = worst subjective health state; 100 = best subjective health state). The HADS‐A/D scale consists of a questionnaire containing 14 questions, with 7 referring to anxiety and seven referring to depression. With the VAS score, the individual patient burden was determined. This psychometric scale ranges from 1 (no burden) to 10 (maximum burden).

SF‐36 and HADS‐A/D scores were compared with data from German reference population studies. For SF‐36 testing, the reference data were provided by the population‐based Heinz Nixdorf Recall Study and the associated MultiGeneration study (Schmermund et al., 2002). Participants were randomly selected from three German cities (i.e., Essen, Bochum, and Mülheim a.d. Ruhr). Individual matching (1:3) was applied to ensure that CCM cases and population‐based controls had the same distribution over strata defined by age (5‐year groups) and sex. For HADS‐A/D testing, reference values were derived from a large population‐based investigation (Hinz & Brähler, 2011).

### 2.4 Statistical analyses

Statistical analysis was performed using SPSS version 22 (IBM Corp.). Data were tested for normal distribution by performing a Shapiro‐Wilk test. For interval‐scaled data, mean values, and standard deviation were calculated, while absolute numbers and valid percent were used for nominal data. The unpaired t‐test (normally distributed data) or the Mann‐Whitney U test (non‐normally distributed data) were used to compare continuous variables. Effect size was reported using Cohen's d, and values were categorized according to an established classification system: small effect (>0.2), medium effect (>0.5), and large effect (>0.8) (Rice & Harris, 2005). The degree of correlation was calculated using the Spearman's rank correlation test. For linear regression analyses, R^2^ values were determined. All tests were two tailed, and *p*‐values < .05 were defined as significant.

## 3 RESULTS

### 3.1 Patient demographics

A total of 37 patients were included in this study. Mean age was 45.8 ± 14.4 years, and 20 participants (54.1%) were female. Six patients (16.2%) exhibited multiple CCMs. Lesions were most commonly located in the temporal lobe. CCM‐related hemorrhage was a common phenomenon and affected 18 (48.6%) individuals. The majority of patients (97.3%) were in good clinical condition (mRS≤2). Fifteen participants (40.4%) had diseases that were not related to CCM disease, including 13 patients (35.1%) suffering from physical and two patients (5.4%) from psychiatric comorbidities. Detailed information on demographic, anatomic, and clinical data is provided in Table 1.

**TABLE 1:** Demographic, anatomic, and clinical characteristics

Characteristic	Frequency
Total number of patients with CRE, *n*	37
Age, years, mean ± SD	45.8 ± 14.4
Sex, *n* (%)	
Male	17 (45.9)
Female	20 (54.1)
CCM number, *n* (%)	
Solitary (1 CCM)	31 (83.8)
Multiple (≥2 CCM)	6 (16.2)
CCM localization, *n* (%)	
Frontal lobe	9 (24.3)
Parietal lobe	5 (13.5)
Temporal lobe	10 (27)
Occipital lobe	3 (8.1)
Multiple lobes	7 (18.9)
Subcortical	3 (8.1)
CCM‐related hemorrhage, *n* (%)	18 (48.6)
mRS, *n* (%)	
0	2 (5.4)
1	31 (83.8)
2	3 (8.1)
3	1 (2.7)
Comorbidities, *n* (%)	
Physical comorbidities	13 (35.1)
Psychiatric comorbidities	2 (5.4)
CRE semiology, *n* (%)	
Focal epilepsy	11 (29.7)
Generalized epilepsy	23 (62.2)
Absence epilepsy	3 (8.1)
No. of seizures, *n* (%)	
1 CCM‐related seizure	24 (64.9)
≥2 CCM‐related seizures	13 (35.1)
Antiepileptic treatment, *n* (%)	
No treatment	9 (24.3)
Monotherapy (1 drug)	22 (59.3)
Combination therapy (2 drugs)	6 (16.2)
Antiepileptic medication, *n* (%)	
Carbamazepine	1 (2.9)
Clonazepam	1 (2.9)
Lacosamide	1 (2.9)
Lamotrigine	5 (14.7)
Levetiracetam	19 (55.9)
Oxcarbazepine	1 (2.9)
Pregabalin	1 (2.9)
Valproate	3 (8.8)
Zonisamide	2 (5.9)
Time between first and last CCM‐related seizure, months, mean ± SD	33.2 ± 88.7
Time between first CCM‐related seizure and survey, months, mean ± SD	60.1 ± 101.5
Time between last CCM‐related seizure and survey, months, mean ± SD	28.9 ± 59.6

Abbreviations: CCM: cerebral cavernous malformation; CRE: cavernoma‐related epilepsy; mRS: modified Rankin Scale; *n*: number; n/a: not applicable; SD: standard deviation.

### 3.2 Cavernoma‐related epilepsy

At the time of testing, all participants had experienced at least one epileptic seizure. Most patients (64.9%) had a history of one single seizure, but several individuals (35.1%) were admitted with a history of recurrent seizures. In the majority of cases (75.7%), anticonvulsant treatment was administered at the time of testing, mainly consisting of one single drug. According to treatment guidelines, the most common medication was levetiracetam (55.9%) and lamotrigen (14.7%). Patients with multiple seizures featured a relatively low seizure frequency. At admission, these patients had a history of 33.2 ± 88.7 months between first and last seizure. In the total cohort, participants were interviewed 60.1 ± 101.5 months after their first seizure and 28.9 ± 59.6 months after their last seizure. Detailed information on clinical data is provided in Table 1.

### 3.3 Health‐related quality of life

**FIGURE 1:**
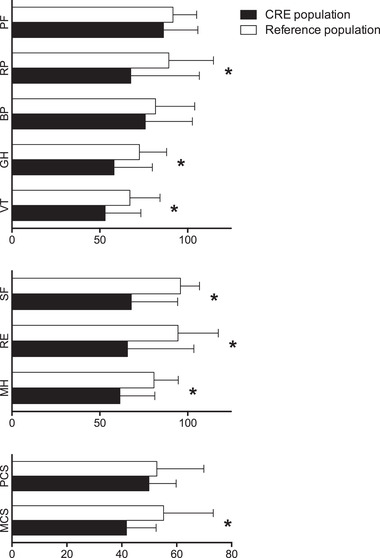
Short Form‐36 (SF‐36) domains including physical health, mental health, and component scores. Physical functioning (PF), social functioning (SF), role limitations due to physical problems (RP), role limitations due to emotional problems (RE), mental health (MH), vitality (VT), bodily pain (BP), and general health perceptions (GH), physical health score (PCS), mental health score (MCS). Asterisks indicate statistical significance compared to reference population (unpaired‐t‐test)

HRQOL was assessed using the German version of the SF‐36. The results for CRE patients and for the age‐ and gender‐matched German reference population are presented in Figure 1. Compared to the reference population, CCM patients with CRE had decreased scores in all domains and component scores. In 18 patients (48.6%), the PCS was 2 points lower than the mean score of the average population. Thirty‐three participants (89.2%) experienced a similar decrease in the MCS. Notably, a two‐point decrease is regarded as relevant (McHorney & Ware, 1995; Ware et al., 1995). Statistical testing demonstrated significantly impaired scores in overall mental health score and in six additional subdomains (*p* < .001). Patients experienced limitations in usual role activities due to physical impairment (RP, *p* = .0001), attenuated general health (GH, *p* = .0001), decreased vitality (VT, *p* = .0001), limitations in social activities because of physical or emotional problems (SF, *p* = .0001), limitations in usual role activities due to emotional problems (RE, *p* = .0001) and worsened mental health (MH, *p* = .0001). Detailed analyses are provided in Table 2. Further investigation revealed differences in physical health component scores of patients with single (52.0 ± 7.7) or multiple (39.9 ± 13.8) CCM (*p* = .004), and differences in mental health component scores of patients with (37.7 ± 11.6) or without (44.9 ± 8.9) history of CCM‐related hemorrhage (*p* = .039). Notably, there were no differences in test performance between male and female individuals.

**TABLE 2:** HRQOL testing in CRE and healthy populations

SF‐36 scale	CRE population, mean ± SD	Reference population, mean ± SD	*p* ^a^	Cohen's d‐value	Cohen's d‐effect^b^
Physical health subdomains					
PF	86.2 ± 19.6	91.6 ± 13.5	.0638	0.40	+
RP	67.6 ± 39.0	89.3 ± 25.4	**.0001**	0.85	+++
BP	75.9 ± 26.8	81.7 ± 22.3	.1954	0.26	+
GH	58.1 ± 21.8	72.5 ± 15.5	**.0001**	2.62	+++
VT	53.0 ± 20.4	67.1 ± 17.2	**.0001**	0.82	+++
Mental health subdomains					
SF	67.9 ± 26.4	95.9 ± 10.8	**.0001**	2.59	+++
RE	65.7 ± 37.8	94.5 ± 22.9	**.0001**	1.26	+++
MH	61.4 ± 19.9	80.8 ± 13.8	**.0001**	1.42	+++
Component scores					
PCS	49.9 ± 9.9	52.8 ± 17.1	.3302	0.17	+
MCS	41.7 ± 10.8	55.2 ± 18.1	**.0001**	0.75	++
Cohort matching					
Age (y), mean ± SD	45.8 ± 14.4	45.8 ± 14.3	n/a	n/a	n/a
Sex, *n* (%)					
Male	17 (45.9%)	51 (45.9%)	n/a	n/a	n/a
Female	20 (54.1%)	60 (54.1%)			

*Note*: Control‐to‐case ratio was 3:1. Significant *p*‐values are in bold font.

Abbreviations: BP, bodily pain; CRE, cavernoma‐related epilepsy; GH, general health perception; MCS, mental health score; MH, Mental health; n/a, data not available; PCS, physical health score; PF, physical functioning; RE, role emotional; RP, role physical; SF, social functioning; VT, vitality.

^a^Two‐tailed Student's t‐test.

^b^Interpretation of d according to Cohen: 0.2– 0.5 small effect size (+), 0.5–0.8 medium effect size (++), > 0.8 large effect size (+++).

### 3.4 Perception of illness, anxiety, and depression

Subjective perception of illness and burden of CRE were evaluated using a visual analog scale (VAS). The mean VAS score was 5.4 ± 2.3, indicating considerable symptom severity. Depression and anxiety were determined using the hospital anxiety and depression rating scale (HADS‐A/D). In the CRE population, the mean HADS‐A score was 7.9 ± 4.4 with 19 patients (51.4%) revealing a score more than or equal to 8, indicating relevant levels of anxiety. Compared to the average German population, values were considerably elevated (Hinz & Brähler, 2011). In contrast to anxiety, depression was less common in CRE patients. The mean HADS‐D score was 5.6 ± 4.2 with 10 patients (27%) scoring 8 or more. These values were comparable to data derived from the German reference population (Hinz & Brähler, 2011). Detailed analyses are listed in Table 3. Notably, HADS‐A, HADS‐D, and VAS scores demonstrated a high degree of concordance among each other, indicating a correlation between the level of anxiety, depression, and perception of illness. Moreover, HADS‐A and HADS‐D were significantly associated with SF‐36 component scores (*p* ≤ .003), except for HADS‐D and PCS. Detailed analyses are provided in Table S1. Subsequent analyses demonstrated differences in HADS‐D scores of patients with (7.3 ± 4.9) or without (4.3 ± 3.2) history of CCM‐related hemorrhage (*p* = .030). Again, there were no differences in test performance between male and female individuals.

**TABLE 3:** Anxiety, depression, and subjective disease burden in CRE, CCM, and healthy populations

Population		HADS‐A			HADS‐D			VAS
		Total	<8	≥8	Total	<8	≥8	Total
		Mean ± SD	*n* (%)	*n* (%)	Mean ± SD	*n* (%)	*n* (%)	Mean ± SD
CRE (*n* = 37)		7.9 ± 4.4	18 (48.6)	19 (51.4)	5.6 ± 4.2	27 (73.0)	10 (27.0)	5.4 ± 2.3
Reference^a^ (*n* = 4410)	Male	4.4 ± 3.3	1580 (81.9)	349 (18.1)	4.8 ± 4.0	1468 (76.1)	461 (23.9)	n/a
	Female	5.0 ± 3.6	1905 (76.8)	576 (23.2)	4.7 ± 3.9	1898 (76.5)	583 (23.5)	n/a

Abbreviations: CRE, cavernoma‐related epilepsy; HADS, hospital anxiety and depression rating scale; n/a, not available; VAS, visual analogue scale.

^a^Data on normative values of the German population according to Hinz & Brähler (2011).

### 3.5 Seizure control and seizure frequency

**FIGURE 2:**
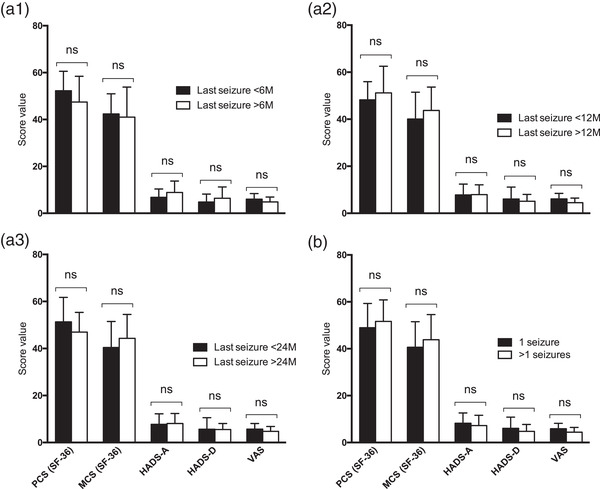
Short Form‐36 (SF‐36) component score, hospital anxiety, and depression rating scale (HADS‐A/D) score and visual analogue scale (VAS) score assessment in patients with seizure control for (A1) 6, (A2) 12, or (A3) 24 or in patients with (B) seizure multiplicity. Unpaired t‐test was applied *Abbreviations*: MCS, mental health score; ns, not significant; PCS, physical health score

In the majority of cases, anticonvulsant medication resulted in sufficient seizure control, but some patients experienced pharmacoresistant epilepsy. We thus investigated the impact of seizure control on HRQOL, anxiety, depression, and perception of illness. Comparison between patients with good seizure control for more or less than 6, 12, or 24 months demonstrated no significant differences in SF‐36 component scores (*p* > .05), HADS‐A/D scores (*p* > .05), or VAS scores (*p* > .05), indicating minor impact of seizure control on quality of life, mood, and illness perception. Detailed values are presented in Figure 2a. Comparable with seizure control, frequency of experienced seizures had no influence on psychometric test results. Values for HRQOL, anxiety, depression, and perception of illness revealed no differences between patients who had experienced one or multiple seizures. The results are provided in Figure 2b.

### 3.6 Time since diagnosis

**FIGURE 3:**
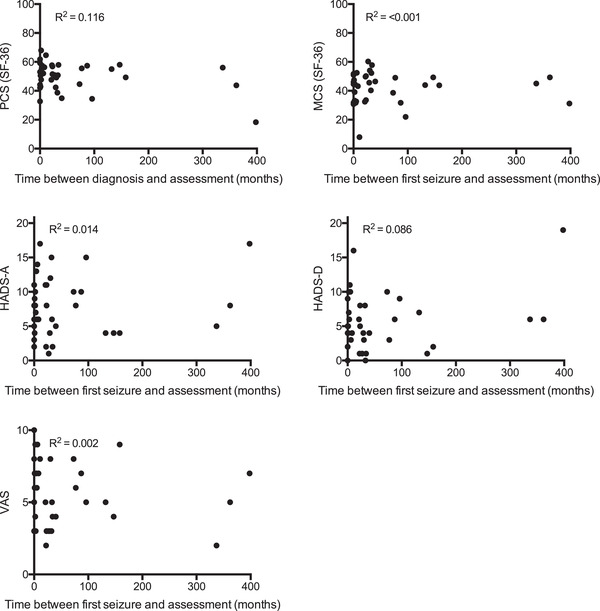
Correlation between time since epilepsy onset and testing and Short Form‐36 (SF‐36) component scores, hospital anxiety, and depression rating scale (HADS‐A/D) scores or visual analogue scale (VAS) scores. Linear regression analyses were applied *Abbreviations*: MCS, mental health score; PCS, physical health score

Due to the cross‐sectional design of this study, patients were examined at different time points. Accordingly, time between first seizure and testing was referred to SF‐36 component scores, HADS‐A/D scores, and VAS scores. Data were visualized in scattered plots, and linear regression analyses revealed low R^2^ values, indicating that none of the variance in test results was explained by the time between first seizure and testing. The results are illustrated in Figure 3.

## 4 DISCUSSION

In this cross‐sectional study, we investigated the impact of medically treated CRE on quality of life and mood in a subset of patients who were not referred to surgery for various reasons. Patient‐reported surveys were used to assess data, and results were related to seizure control.

Although seizure diseases are one of the most common presentations in CCM patients (Rosenow et al., 2013), functional and psychosocial burden is rarely discussed. Moreover, literature dealing with quality of life in CRE is exclusively limited to studies investigating the outcome after CCM removal (Dammann, Wrede, et al., 2017; Ruan et al., 2015; Van Gompel et al., 2010). Since treatment is often personalized, particularly in the case of CRE, patients and surgeons often face the difficult question of whether to opt for surgery or continue drug treatment (Dammann et al., 2021). Recently published data have highlighted the value of open and minimally invasive surgery for epileptogenic CCM lesions, including pediatric and adult patients (Dammann, Wrede, et al., 2017; Kapadia et al., 2021; Lee et al., 2017; Lin et al., 2018; Ozlen et al., 2021; Satzer et al., 2020; Schuss et al., 2020; Willie et al., 2019). Nevertheless, although early CCM removal is often feasible and effective, this topic is still a matter of debate, and high‐quality evidence‐based recommendations are missing (Awad & Polster, 2019; Zanello et al., 2019). In this scenario, clinical decision‐making should not only rely on neurological assessment, due to the following reasoning.

Recently, Herten et al. (2021) tested quality of life in noninvasively treated patients and concluded that CCM strongly decreases HRQOL, even in the absence of functional impairment or neurological symptoms. Moreover, while numerous authors have described the negative impact of epileptic seizures on quality of life (Mahrer‐Imhof et al., 2013; Villanueva et al., 2013), the impact of CRE in CCM patients has yet not been considered. To this end, we present a novel study investigating quality of life, anxiety, depression, and illness perception in medically treated CRE patients. In this trial, we were able to confirm the negative impact of epileptic seizures on quality of life and mood, even though the rating of burden showed pronounced interindividual differences. Furthermore, the calculated effect sizes ranged from small to large, underlining the high degree of heterogeneity in patient burden. Interestingly, neither the number of seizures experienced nor the length of time with sufficient seizure control had an influence on the patient‐reported outcome. One might speculate that it is the diagnosis of CRE, but not the extent of neurological impairment, which is the most important contributing factor to the reduced quality of life and mood observed in our study.

In light of the promising seizure outcome after surgery for CRE (Dammann, Wrede, et al., 2017; Rosenow et al., 2013) and the increasing HRQOL after CCM removal in general (Cornelius et al., 2016; Dukatz et al., 2011), one might hypothesize that patients with strongly decreased quality of life could be eligible for lesionectomy. Patient‐reported outcome parameters, for example, testing with SF‐36 or HADS‐A/D, could be used as benchmarks to assess the individual degree of functional and psychosocial deficits and to improve clinical management.

The shortcomings of this study are mainly related to the small sample size, the missing disease specificity of test instruments, and the reporting on data of a previously published patient cohort. Since symptomatic CCMs are often referred to surgery, the number of drug‐treated CRE patients remains low. However, compared to large studies on CCM, our cohort exhibits typical characteristics with similar baseline data (Al‐Shahi Salman et al., 2012; Horne et al., 2016). Thus, patients included in this study can be regarded as a representative sample of CCM patients, increasing the external validity of our reported results. Notably, SF‐36, HADS‐A/D, and VAS scores were not established to assess burden in CCM or CRE patients, limiting the specificity and sensitivity of testing. Nevertheless, disease‐specific test instruments are missing, and SF‐36, HADS‐A/D, and VAS are established, validated, and convenient. These parameters might reflect disease burden beyond neurological impairment or seizures, making them useful for clinical application. Since parts of the data were published previously in a trial investigating HRQOL in patients with untreated cavernous malformations of the central nervous system, novelty of data is limited.

## 5 CONCLUSION

Our report uncovers previously unrecognized functional and psychosocial deficits in medically treated CRE patients and highlights the clinical importance of psychometric testing. Seizure control had no restoring effect on outcome, underlining the value of surgical CCM removal irrespective of sufficient anticonvulsant treatment. A systematic analysis in a larger cohort is warranted.

## CONFLICT OF INTEREST

The authors have declared that no competing interest.

## AUTHOR CONTRIBUTIONS

**Laurèl Rauschenbach**: responsible for designing the study protocol, summarized data, provided data, conducted data analysis, interpreted results, created figures and tables, wrote the manuscript, and reviewed the manuscript. **Pauline Bartsch**: summarized data, provided data, conducted data analysis, created figures and tables, and reviewed the manuscript. **Alejandro N. Santos**: summarized data, provided data, and reviewed the manuscript. **Annika Lenkeit**: summarized data, provided data, and reviewed the manuscript. **Marvin Darkwah Oppong**: summarized data, provided data, interpreted results, and reviewed the manuscript. **Karsten H. Wrede**: responsible for designing the study protocol and reviewed the manuscript. **Ramazan Jabbarli**: responsible for designing the study protocol and reviewed the manuscript. **Witold X. Chmielewski**: responsible for designing the study protocol, conducted data analysis, interpreted results, and reviewed the manuscript. **Börge Schmidt**: responsible for designing the study protocol, conducted data analysis, interpreted results, and reviewed the manuscript. **Carlos M. Quesada**: interpreted results and reviewed the manuscript. **Michael Forsting**: provided data, reviewed the manuscript. **Ulrich Sure**: responsible for designing the study protocol, provided data, interpreted results, and reviewed the manuscript. **Philipp Dammann**: responsible for designing the study protocol, summarized data, provided data, interpreted results, wrote the manuscript and reviewed the manuscript.

### PEER REVIEW

The authors of this study thank the anonymous reviewers for their careful reading and improving this work. The peer review history for this article is available at https://publons.com/publon/10.1002/brb3.2595.

## Supporting information

Supporting Information

## Data Availability

The data that support the findings of this study are available in the supplementary material of this article and from the corresponding author upon reasonable request.
