# Supplementary material for: Quality of life and mood assessment in conservatively treated cavernous malformation‐related epilepsy
Source: Brain Behav. 2022 Apr 25;12(6):e2595. doi: 10.1002/brb3.2595 (PMC9226805; doi:10.1002/brb3.2595)
Supplement: Supplementary file 1 — Supporting Information [file BRB3-12-e2595-s001.docx]

**Supplemental material:**

| **Score combination** | **Correlation (r, p-value)** | |
| --- | --- | --- |
|  | **r value** | **p value** |
| HADS-A / HADS-D | 0.73 | **<0.001** |
| HADS-A / VAS | 0.49 | **0.002** |
| HADS-D / VAS | 0.36 | **0.03** |
| PCS (SF-36) / HADS-A | -0.74 | **<0.001** |
| PCS (SF-36) / HADS-D | -0.07 | 0.68 |
| PCS (SF-36) / VAS | -0.05 | 0.77 |
| MCS (SF-36) / HADS-A | -0.48 | **0.003** |
| MCS (SF-36) / HADS-D | -0.74 | **<0.001** |
| MCS (SF-36) / VAS | -0.24 | 0.17 |
| **Supplemental Table 1. Correlation between HADS-A, HADS-B, VAS and SF-36 (PCS, MCS) testing in CRE patients.** The r and p values were calculated with the Spearman’s rank correlation test (r = correlation coefficient). Boldface type illustrates statistical significance. | | |
